# The age distribution of mortality from novel coronavirus disease (COVID-19) suggests no large difference of susceptibility by age

**DOI:** 10.1038/s41598-020-73777-8

**Published:** 2020-10-06

**Authors:** Ryosuke Omori, Ryota Matsuyama, Yukihiko Nakata

**Affiliations:** 1 Research Center for Zoonosis Control, Hokkaido University, Kita-20 Nishi-10, Kita-Ku, Sapporo, Hokkaido 001-0020 Japan; 2 Graduate School of Biomedical and Health Sciences, Hiroshima University, Hiroshima, Japan; 3 Department of Physics and Mathematics, Aoyama Gakuin University, Tokyo, Japan

**Keywords:** Viral infection, Epidemiology, Viral infection, Epidemiology

## Abstract

Among Italy, Spain, and Japan, the age distributions of COVID-19 mortality show only small variation even though the number of deaths per country shows large variation. To understand the determinant for this situation, we constructed a mathematical model describing the transmission dynamics and natural history of COVID-19 and analyzed the dataset of mortality in Italy, Spain, and Japan. We estimated the parameter which describes the age-dependency of susceptibility by fitting the model to reported data, including the effect of change in contact patterns during the epidemics of COVID-19, and the fraction of symptomatic infections. Our study revealed that if the mortality rate or the fraction of symptomatic infections among all COVID-19 cases does not depend on age, then unrealistically different age-dependencies of susceptibilities against COVID-19 infections between Italy, Japan, and Spain are required to explain the similar age distribution of mortality but different basic reproduction numbers (*R*_0_). Variation of susceptibility by age itself cannot explain the robust age distribution in mortality by COVID-19 infections in those three countries, however it does suggest that the age-dependencies of (i) the mortality rate and (ii) the fraction of symptomatic infections among all COVID-19 cases determine the age distribution of mortality by COVID-19.

## Introduction

Since its emergence, coronavirus disease 2019 (COVID-19) has resulted in a pandemic and has produced a huge number of cases worldwide^1^. As of May 29, 2020, the number of confirmed cases in Italy was 382.3 (per 100,000 population), with 507.2 in Spain, and 13.2 in Japan^1^. Of those infected, it has been reported that elderly individuals account for a large portion of fatal cases inducing a large heterogeneity in the age distribution of mortality^2–4^.

The expected value of mortality (the number of deaths, hereafter referred to as mortality) is calculated as the product of the number of cases and the mortality rate among cases (hereafter referred to as morality rate). As the background mechanism of the heterogeneity of mortality by age, the association of two epidemiological factors with mortality can be considered: (i) the age-dependency of susceptibility to infection, which is related to the heterogeneity in the number of cases, and (ii) the age-dependency of severity, which is related to the heterogeneity in the mortality rate, e.g. the rate of becoming a symptomatic, severe, or fatal case among infected individuals. For the first factor, a high susceptibility for infection will generate a larger number of infections and result in an increase in fatal cases. The possibility of heterogeneity in susceptibility by age was pointed out by the analysis of epidemiological data reported from Wuhan, China^4–6^ and from Iceland^7^. For the second factor, an increase in severity will result in a higher mortality rate and subsequently a rise in the number of fatal cases. This assumption is also reasonable because elder age as well as the existence of comorbidities, which are likely with aging, have been reported as risk factors for severe COVID-19 infections^8–13^. Although not yet shown in relation to severe acute respiratory syndrome coronavirus 2 (SARS Cov-2), which is the causal agent of COVID-19, the presence of age-dependent enhancement of severity has been suggested in SARS coronavirus by the analysis of the innate immune responses in the BALB/c mouse model^14–16^. Additionally, it has been suggested that antibody-dependent enhancement (ADE) can contribute to the formation of the observed age-dependency of severity, as suggested in SARS and Middle East respiratory syndrome (MERS) cases^17–22^.

**Figure 1:**
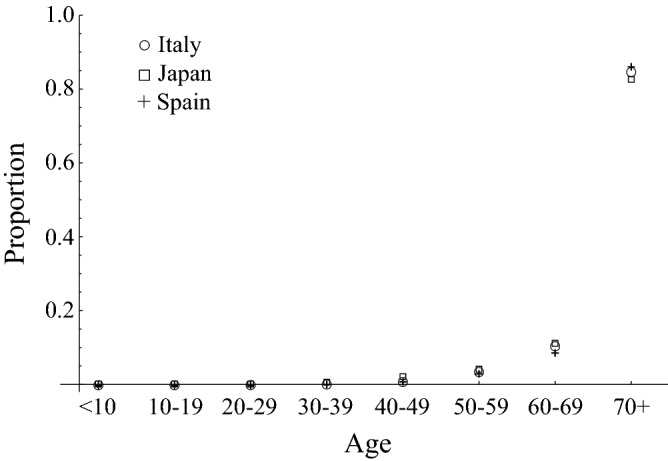
The age distribution of mortality by COVID-19 in Italy reported on 13th May 2020, Japan reported on 7th May 2020, and Spain reported on 12th May 2020. Circle, square, and “+” denote Italy, Japan, and Spain.

Interestingly, the age distribution of mortality by COVID-19 (the distribution of the proportion of deaths per age group among all deaths), is similar between Italy, Japan, and Spain, even though the number of deaths are quite different among them^23–25^ (Fig. 1). The reported number of deaths was 3 in 0–9 years old (yo), 0 in 10–19 yo, 11 in 20–29 yo, 58 in 30–39 yo, 257 in 40–49 yo, 1,051 in 50–59 yo, 3,107 in 60–69 yo, and 25,038 in 70 + yo in Italy as of May 13, 2020. In Japan, that was 0 in 0–9 yo, 0 in 10–19 yo, 0 in 20–29 yo, 2 in 30–39 yo, 8 in 40–49 yo, 16 in 50–59 yo, 44 in 60–69 yo, and 330 in over 70 + yo as of May 7, 2020. In Spain, that was 2 in age 0–9 yo, 5 in 10–19 yo, 23 in 20–29 yo, 61 in 30–39 yo, 198 in 40–49 yo, 607 in 50–59 yo, 1669 in 60–69 yo, and 16,253 in over 70 + yo as of May 12, 2020. According to projections by the United Nations^26^, the population size for 2020 per 1,000,000 was 4.99 in 0–9 yo, 5.73 in 10–19 yo, 6.10 in 20–29 yo, 7.00 in 30–39 yo, 9.02 in 40–49 yo, 9.57 in 50–59 yo, 7.48 in 60–69 yo, and 10.55 in 70 + yo in Italy. In Japan, that was 10.18 in 0–9 yo, 11.27 in 10–19 yo, 12.15 in 20–29 yo, 14.46 in 30–39 yo, 18.47 in 40–49 yo, 16.54 in 50–59 yo, 15.88 in 60–69 yo, and 27.54 in 70 + yo. In Spain, that was 4.23 in 0–9 yo, 4.74 in 10–19 yo, 4.62 in 20–29 yo, 5.90 in 30–39 yo, 7.94 in 40–49 yo, 7.05 in 50–59 yo, 5.34 in 60–69 yo, and 6.94 in 70 + yo. The large difference in the number of deaths between the countries suggests a large difference in their basic reproduction numbers, *R*_0_s. An independency between age distribution of mortality by COVID-19 and *R*_0_ is suggested. From this independency of age distributions of mortality from *R*_0_, it can be expected that the contribution of heterogeneity in susceptibility by age to forming the age distribution of mortality is small. That is because, as we will show in this paper, though the age-dependency of severity will naturally produce a proportional effect on the distribution of mortality and result in the formation of robust distributions, when the age-dependency of susceptibility forms the age distribution of mortality, the age distribution of mortality highly depends on *R*_0_ and shows variability.

To understand the background of robust age distribution of mortality with varied *R*_0_, we constructed a mathematical model describing the transmission dynamics of COVID-19 and analyzed the impact of age-dependent susceptibility on the age distribution of mortality. The heterogeneity in social contacts by age may also contribute to the age distribution of mortality. Our model took into account the heterogeneity in social contacts by age and country, and the effect of behavioral change outside of the household during the outbreak. We also estimated and compared the age-dependent susceptibility in Japan, Italy, and Spain to argue the existence of heterogeneity in susceptibility among age groups.

## Results

**Figure 2:**
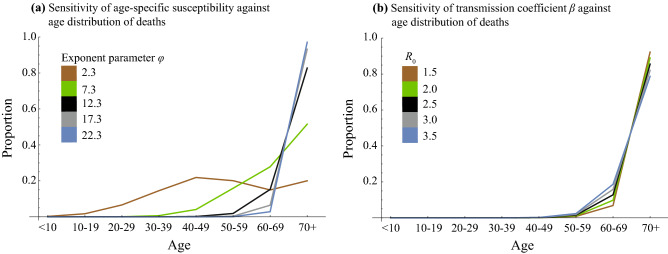
The sensitivity of (**a**) age-dependency of susceptibility and (**b**) transmission coefficient *β* against age distribution of mortality when age-independent mortality was assumed. In panel (**a**), all parameters except the exponent parameter *φ*, describing the variation of susceptibility among age groups, were fixed and parameterized as *R*_0_ = 2.9 in the setting for Spain. In panel (**b**), all parameters parameterized as the setting for Spain (*φ* = 12.3) were fixed except transmission coefficient *β*.

**Figure 3:**
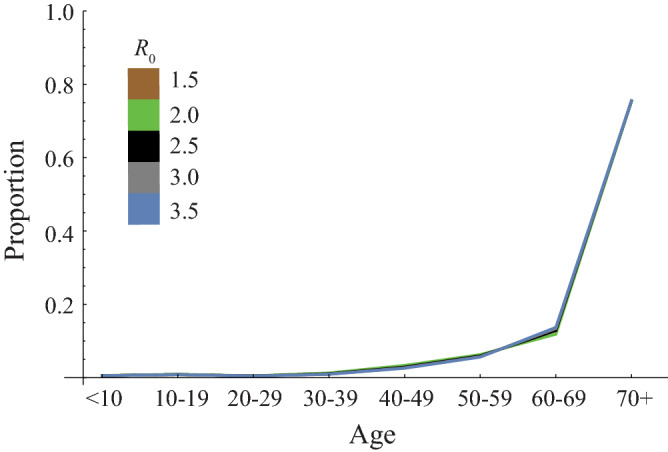
The sensitivity of transmission coefficient *β* against age distribution of mortality when it was assumed that age-dependent mortality was proportional to cCFR per age group. All parameters were fixed and parameterized as the setting for Spain except the transmission coefficient *β*.

Our result shows variation of susceptibility among age groups measured by the exponent parameter *φ* can explain the age distribution of mortality by COVID-19 (Fig. 2a). However, the age distribution of mortality formed by the age-dependency of susceptibility is influenced by the value of *R*_0_ (Fig. 2b), which cannot explain the similarity in age distributions of mortality among Italy, Japan, and Spain. On the other hand, if susceptibility is constant among age groups, the impact of *R*_0_ is quite small on the age distribution of mortality (Fig. 3).

**Figure 4:**
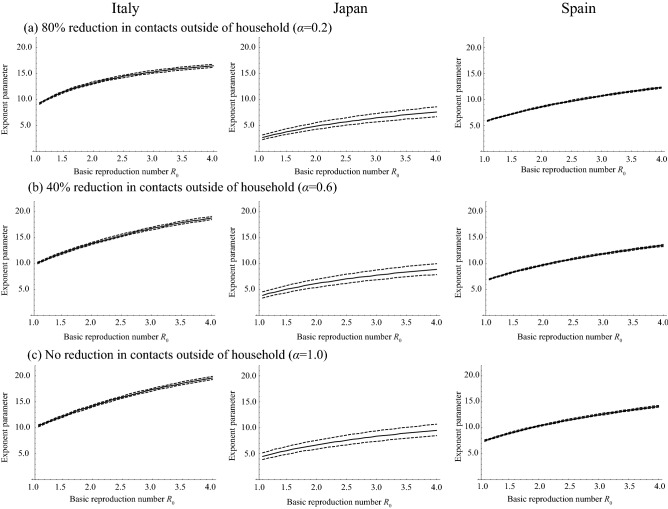
The estimate of exponent parameter *φ* describing the variation of susceptibility among age groups using model 1 and assuming that mortality rate does not depend on age. True and broken lines represent the maximum likelihood estimates and 95% confidence intervals, respectively.

Assuming that the age-dependency of mortality by COVID-19 is determined by only age-dependent susceptibility (model 1), i.e., the mortality rate does not depend on age, the exponent parameter, *φ*, describing the variation of susceptibility among age groups for each country, Italy, Japan, and Spain, was estimated as shown in Fig. 4. From the difference of the *R*_0_ value and country, the estimated value of *φ* is largely varied. The impact of reductions in contacts outside of the household on the estimated value of *φ* was small. The estimate of *φ* in Italy, assuming a range of *R*_0_ = 2.4–3.3^27,28^ was 15.0 (95% CI 14.0–16.0), 16.3 (95% CI 14.9–17.7), and 16.9 (95% CI 15.4–18.4) for 80%, 40%, and no reduction in contacts outside of the household. For Japan, the estimate of *φ* assuming *R*_0_ = 1.7^29^ was 4.2 (95% CI 3.7–4.9), 5.5 (95% CI 4.9–6.3), and 6.1 (95% CI 5.4–6.9) for 80%, 40%, and no reduction in contacts outside of the household. When it comes to Spain, the estimate of *φ* assuming an *R*_0_ = 2.9^30^ was 10.5 (95% CI 10.4–10.6), 11.7 (95% CI 11.6–11.9), and 12.3 (95% CI 12.2–12.5) for 80%, 40%, and no reduction in contacts outside of the household.

**Figure 5:**
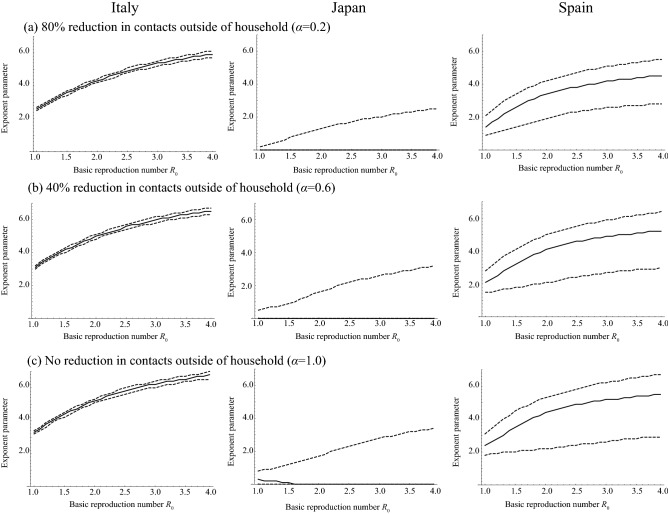
The estimate of exponent parameter *φ* describing the variation of susceptibility among age groups using model 2 and assuming that the fraction of infections that becomes symptomatic among all COVID-19 cases is 0.25. True and broken lines represent the maximum likelihood estimates and 95% confidence intervals, respectively.

The estimates of *φ,* assuming that the mortality by COVID-19 infections depends on age but the fraction of infections becoming symptomatic does not depend on age (model 2), were also varied by the value of *R*_0_ and by country (Fig. 5, s1 and s2). Employing the same assumptions of *R*_0_ value, the estimate of *φ* in Italy was 5.2 (95% CI 4.7–5.7), 5.9 (95% CI 5.3–6.4), and 6.1 (95% CI 5.5–6.6) for 80%, 40%, and no reduction in contacts outside of the household. For Japan, the estimate of *φ* was 0.0 (95% CI 0.0–1.0), 0.0 (95% CI 0.0–1.2), and 0.0 (95% CI 0.0–1.4) for 80%, 40%, and no reduction in contacts outside of the household. For Spain, the estimate of *φ* was 4.1 (95% CI 2.5–5.0), 4.8 (95% CI 2.6–5.8), and 5.1 (95% CI 2.6–6.2) for 80%, 40%, and no reduction in contacts outside of the household.

## Discussion

In the present study, we explored the role of susceptibility to COVID-19 in explaining the age distribution of mortality by COVID-19. Interestingly, the age distributions of mortality from COVID-19 are quite similar between Italy, Japan, and Spain (Fig. 1). When comparing the age distributions of mortality, only the comparison between Italy and Spain is significant (*p* < 0.05 in Wilcoxon rank sum test with Bonferroni correction). On the other hand, the numbers of deaths are quite different (29,525 for Italy, 400 for Japan, 18,818 for Spain). Indeed, *R*_0_ values are largely different: 2.4–3.3 for Italy^27,28^, 1.7 for Japan^29^, and 2.9 for Spain^30^. If the variation of mortality by age is determined by only the age-dependency of susceptibility, the age distribution of mortality is affected by *R*_0_ as shown in Fig. 2b. However, we observed a similarity in age distributions of mortalities between Italy, Japan, and Spain where their *R*_0_s are quite different. Indeed, unrealistically different *φ*s among these three countries are required to explain their age distribution of mortality for both settings, (i) age-independent mortality, and, (ii) the fraction of infections that becomes symptomatic among all COVID-19 cases, *f*_*s*_, does not depend on age. Although we cannot fully reject the existence of age-dependency in susceptibility, our results suggest that it does not largely depend on age, but rather that age-dependency in severity highly contributes to the formation of the observed age distribution in mortality.

The estimates of *φ*s assuming age independency in symptomatic infections were smaller than those that assumed age independency in mortality. This suggests that the age-dependency of the confirmed case fatality rate (cCFR), which can be biased by the age-dependent difference of the fraction of symptomatic infections among all cases, partially explains the age distribution in mortality. Indeed, when we assumed that the fraction of symptomatic infections was not dependent on age, the estimate of *φ* in Japan was close to zero in all scenarios regarding the fraction of symptomatic infections, meaning that susceptibility is constant among age groups (Fig. 5). Although we observed *φ*s not close to zero in Italy and Spain, this does not mean straightforwardly that susceptibility is age dependent because there is room for an alternative explanation: not susceptibility, but an age-dependent fraction of symptomatic infections can explain this age-dependency. Unfortunately, as we do not yet have detailed data regarding the age-dependent fraction of symptomatic infections and the rate of diagnosis in COVID-19, we cannot conclude which factors (i.e., susceptibility or the fraction of symptomatic infection among all cases) contributed to the observed age-dependency.

Wu et al.^4^ showed variation of susceptibility to symptomatic infection by age. This susceptibility can be expressed as the product of the susceptibility and the fraction of symptomatic infection among all cases. To accurately understand susceptibility (i.e., without the constraint of the symptom onset), estimates of the age-dependent fraction of symptomatic infections is required.

To understand the mechanism of age-dependency of mortality by COVID-19, an accurate age-dependent mortality rate is required. The data of mortality by COVID-19 infections used in this study might not cover all mortalities by COVID-19 infections. To estimate the age-dependent mortality rate, an accurate estimate of the case fatality rate is required. However, the number of cases, which is the denominator of the case fatality rate, is difficult to estimate for COVID-19 due to changes in the testing rate^31–33^, the change of case definition^34^, selection biases^35^, and the delay between the onset of symptoms and death^12,36–38^ as were the cases we experienced in the surveillance of other emerging diseases ^39,40^. To address this problem, implementation of active epidemiological surveillances, such as a large-scale cohort study including real-time detection of infections, should be considered.

From the modelling perspective on mortality by Covid-19, age-dependency of severity should be carefully taken into consideration. In particular, in the mathematical models of ADE, previous models employed three types of assumptions^41^, the assumption of: increasing susceptibility to infection^42,43^, increasing transmissibility once infection occurred^42,44,45^, and increasing severity and/or mortality associated with infection^46^. Based on our results and from the biological/epidemiological observations of past SARS and MERS cases, the “increasing severity” assumption should be taken into account when analyzing SARS Cov-2 epidemics.

We modelled the age-specific susceptibility as a power law function based on the monotonic increase of mortality by COVID-19 over age as seen in Fig. 1. The power law function is widely used to model heterogeneity, e.g., the heterogeneity in risks of sexually transmitted infections^47^. Although our model for age-specific susceptibility covers a wide variation of monotonic changes, our results might be biased by this formulation if the susceptibility changes over age in non-monotonic fashion.

The increase in width of the confidence interval for the estimate of *φ* by increasing *R*_0_ values were observed in Fig. 5. To explain with the “left-skewed” age-distribution of mortality with high *R*_0_, a large *φ* is required since the higher *R*_0_ value decreased the heterogeneity of mortality by age (Fig. 2b) and the large *φ* increased the heterogeneity of mortality (Fig. 2a). The sensitivity of *φ* to the age-distribution of mortality becomes smaller when *φ* is larger (Fig. 2a), the large widths of the confidence intervals for the estimate of *φ* is necessary to explain the age-distribution of mortality when *R*_0_ is high.

In conclusion, the contribution of age-dependency to susceptibility is difficult to use to explain the robust age distribution in mortalities by COVID-19, and it suggests that the age-dependencies of the mortality rate and the fraction of symptomatic infections among all COVID-19 cases determine the age distribution in mortality from COVID-19. Further investigations regarding age-dependency on the fraction of infections becoming symptomatic is required to understand the mechanism behind the mortality by COVID-19 infections.

## Materials and methods

### Data

We analyzed the number of mortalities caused by COVID-19 in Italy reported on 13th May 2020, Japan reported on 7th May 2020, and Spain reported on 12th May 2020. The data were collected from public data sources in each country^23–25^.

### Model

#### A simple SEIRD model taking into account mixing between age groups (model 1)

To understand the background of robust age distribution of mortality with varied *R*_0_, we employed a mathematical model describing transmissions of COVID-19. Clinical observations suggest that both asymptomatic and symptomatic cases are infectious after the latent period^48,49^, we used a simple age-structured SEIRD (susceptible-exposed-infectious-recovered-dead) model, which can be written as;

1 $${{S}^{^{\prime}}}_{n}=-\beta {\sigma }_{n}{S}_{n}\left(\sum_{m}{k}_{n,m}{I}_{m}\right),$$

2 $${{E}^{^{\prime}}}_{n}=\beta {\sigma }_{n}{S}_{n}\left(\sum_{m}{k}_{n,m}{I}_{m}\right)-\varepsilon {E}_{n},$$

3 $${{I}^{^{\prime}}}_{n}=\varepsilon {E}_{n}-(\gamma +\delta ){I}_{n},$$

4 $${{R}^{^{\prime}}}_{n}=\gamma {I}_{n},$$

5 $${{D}^{^{\prime}}}_{n}=\delta {I}_{n},$$

where *S*_*n*_, *E*_*n*_, *I*_*n*_, *R*_*n*_ and *D*_*n*_ represent the proportion of susceptible, latent, infectious, recovered and dead among the entire population, and the subscript index *n* denotes age group. We stratified the entire population by into eight groups, *n* = 1, 2, 3, 4, 5, 6, 7, and 8 for < 10 yo, 10–19 yo, 20–29 yo, 30–39 yo, 40–49 yo, 50–59 yo, 60–69 yo, and 70 + yo. *β*, *k*_n,m_, *ε*, *γ* and *δ* represent a transmission coefficient, an element of the contact matrix between age group *n* and *m*, the progression rate from latent to infectious, recovery rate and mortality rate by COVID-19 infections, respectively. *σ*_n_ denotes the susceptibility of age group *n*. For the sake of simplicity, based on the short study duration of COVID-19 epidemics compared to the length of a human lifespan, births and deaths from causes other than COVID-19 were ignored. To take into account the effect of behavioral changes outside of the household during the outbreak, *k*_n,m_ is decomposed by a matrix for contacts within household *k*_in,n,m_ and that for contacts outside the household *k*_out,n,m_;

6 $${k}_{n,m}={k}_{in,n,m}+{\alpha k}_{out,n,m},$$

where *α* denotes the reduced fraction of contacts outside of the household. We modelled age specific susceptibility as

7 $${\sigma }_{n}=c{n}^{\varphi }.$$

where *c* is susceptibility among age group 1 and a constant among all age groups, *φ* denotes the exponent parameter describing the variation of susceptibility among age groups. An increase in *φ* means an increase in the variation of susceptibility among age groups, and *φ* = 0 means that susceptibility is equal among all age groups.

#### SEIRD model taking into account mixing between age groups, asymptomatic/symptomatic, and age-dependency of mortality by COVID-19 (model 2)

Model 1 does not classify the cases into asymptomatic and symptomatic cases explicitly. If the progression of disease is largely different between asymptomatic and symptomatic cases, the estimates using model 1 can be biased. In addition, the age-dependency of mortality by COVID-19 infections is not taken into account. Model 2 takes into account both the different progression of disease between asymptomatic and symptomatic cases and the age-dependency of mortality by COVID-19 infections;

8 $${{S}^{^{\prime}}}_{n}=-\beta {\sigma }_{n}{S}_{n}\left(\sum_{m}{k}_{n,m}{I}_{m}\right),$$

9 $${{E}^{^{\prime}}}_{n}=\beta {\sigma }_{n}{S}_{n}\left(\sum_{m}{k}_{n,m}{I}_{m}\right)-\varepsilon {E}_{n},$$

10 $${{I}^{^{\prime}}}_{s, n}=\varepsilon {f}_{s}{E}_{n}-({\gamma }_{s}+{\delta }_{n}){I}_{s,n},$$

11 $${{I}^{^{\prime}}}_{a, n}=\varepsilon {(1-f}_{s}){E}_{n}-{\gamma }_{a}{I}_{a,n},$$

12 $${{R}^{^{\prime}}}_{n}={{\gamma }_{s}{I}_{s,n}+\gamma }_{a}{I}_{a,n},$$

13 $${{D}^{^{\prime}}}_{n}={\delta }_{n}{I}_{s,n},$$

where *I*_s,n_ and *I*_a,n_ represent the proportion of symptomatic and asymptomatic cases among age group *n*. Other compartments are the same as model 1. *f*_*s*_ represents the fraction of symptomatic infections among all COVID-19 cases and *δ*_n_ represents the mortality rate by COVID-19 infection among age group *n*. ${\gamma }_{s}$ and ${\gamma }_{a}$ denote the recovery rates among symptomatic and asymptomatic cases. Other parameters are the same as model 1.

### Parameterizations

We parameterized *ε* and *γ* using values from a previous modelling study of COVID-19^48,50,51^. The average length of the latent period (i.e., 1/ε) was set to 6.4 days^48,50^, assuming that the latent period is equal to the incubation period, and the average length of the infectious period (i.e., 1/γ) was 7 days^48,51^ for model 1. In model 2, to take into account the different infectious period between symptomatic and asymptomatic infections, we set an average length of infectious period among asymptomatic cases (i.e., 1/γ_a_) as 9 days^49^ and an average length of infectious period among symptomatic cases (i.e., 1/γ_s_) as 7 days. We referred to the contact matrices for Italy, Japan, and Spain from Prem et al.^52^. *β* and *c* were controlled such that the basic reproduction number, *R*_0_, becomes arbitral values. *R*_0_ was calculated by constructing a next generation matrix^53,54^ using each country’s demographic data obtained from a public data source^26^.

In terms of parameterization for mortality rate by COVID-19 infection, a reliable estimate of *δ*_*n*_ for COVID-19 is difficult to obtain. Due to the uncertainty of the fraction of symptomatic infections per age group, *δ*_*n*_ is difficult to estimate from observed data, i.e., the confirmed case fatality rate among age group *n* (cCFR_*n*_). Since an estimate of *δ*_*n*_ is difficult to obtain, we employed two different settings (i) *δ*_*n*_ is assumed to be a constant among all age groups as assumed in the model 1, i.e., *δ*_*n*_ = *δ* for any age group *n*, or, (ii) *δ*_*n*_ is calculated from cCFR_*n*_ assuming that the fraction of symptomatic infections among all COVID-19 cases (*f*_*s*_) is not dependent on age as assumed in model 2.

In the setting for model 1, the value of *δ* is not required to estimate *D*_n_ once the value of *R*_0_ is given. We calculated *D*_n_ by calculating the proportions of recovered persons per age group among all recovered persons ${R}_{n}(\infty )/\sum_{n}{R}_{n}(\infty )$ instead of ${D}_{n}(\infty )/\sum_{n}{D}_{n}(\infty )$. In our model, shown in Eq. (1–5), ${R}_{n}(\infty )/\sum_{n}{R}_{n}(\infty )$ is determined by the value of *R*_0_ completely when all parameter values other than *β* and *δ* are fixed, and ${D}_{n}(\infty )/\sum_{n}{D}_{n}(\infty )={R}_{n}(\infty )/\sum_{n}{R}_{n}(\infty )$ if ${\delta }_{n}\ne 0$. The proof can be found in the Supplemental text.

The assumption in model 1, *δ*_*n*_ is constant among all age groups, may be too strong for the COVID-19 epidemic. To take into account the age-dependency of mortality by COVID-19, *δ*_*n*_ was calculated from the cCFR_*n*_ assuming that *f*_*s*_ is not dependent with age. For the setting in model 2, assuming three scenarios; *f*_*s*_ = 0.05, 0.25, and 0.5, *δ*_*n*_ for each country were calculated using cCFR_*n*_ in each country. We obtained *δ*_*n*_ by solving cCFR_*n*_ = *δ*_*n*_/ (*δ*_*n*_ + *γ*_*s*_).

### Fitting

We calculated the proportions of deaths in the age group *n* among all deaths, *D*_n_ (${=D}_{n}(\infty )/\sum_{n}{D}_{n}(\infty )$), and fitted them to the observed data in each country. We solved model 1 shown in Eqs. (1–5) and model 2 shown in Eqs. (8–13) numerically, and *D*_n_ was calculated after sufficient time was given to finish the epidemics. We estimated *φ* using a log likelihood function describing the multinomial sampling process of deaths per age group;

14 $$\sum_{n}{D}_{n}\mathrm{log}\left[{d}_{n}(\varphi )\right]$$

Maximum likelihood estimates of *φ* with given *R*_0_ were obtained by maximizing Eq. (14) and the profile likelihood-based confidence intervals were computed.

## Supplementary information

Supplementary file1

## Data Availability

All data collected and analyzed during this study are included in this published article and its Supplementary Information files.
