# Supplementary material for: The age distribution of mortality from novel coronavirus disease (COVID-19) suggests no large difference of susceptibility by age
Source: Sci Rep. 2020 Oct 6;10:16642. doi: 10.1038/s41598-020-73777-8 (PMC7538918; doi:10.1038/s41598-020-73777-8)
Supplement: Supplementary file 1 — Supplementary file1 [file 41598_2020_73777_MOESM1_ESM.pdf]

Supplemental file

Title: The age distribution of mortality from novel coronavirus disease (COVID-19) suggests no large difference of susceptibility by age

Authors: Ryosuke Omori[1], Ryota Matsuyama[2], Yukihiko Nakata[3]

Affiliations:

[1] Research Center for Zoonosis Control, Hokkaido University, Kita-ku, Sapporo-shi, Hokkaido 001-0020, Japan

[2] Graduate School of Biomedical and Health Sciences, Hiroshima University

[3] Department of Physics and Mathematics, Aoyama Gakuin University

(Correspondence to Ryosuke Omori at: Address: Kita-20 Nishi-10, Kita-Ku, Sapporo, 001-0020, Japan; Tel: +81-11-706-9488, Fax: +81-11-706-9491; Email: omori@czc.hokudai.ac.jp)

Supplemental figures

Figure s1: The estimate of exponent parameter φ describing the variation of susceptibility among age groups using model 2 and assuming that the fraction of infections that becomes symptomatic among all COVID-19 cases is 0.5. True and broken lines represent the maximum likelihood estimates and 95% confidence intervals, respectively.

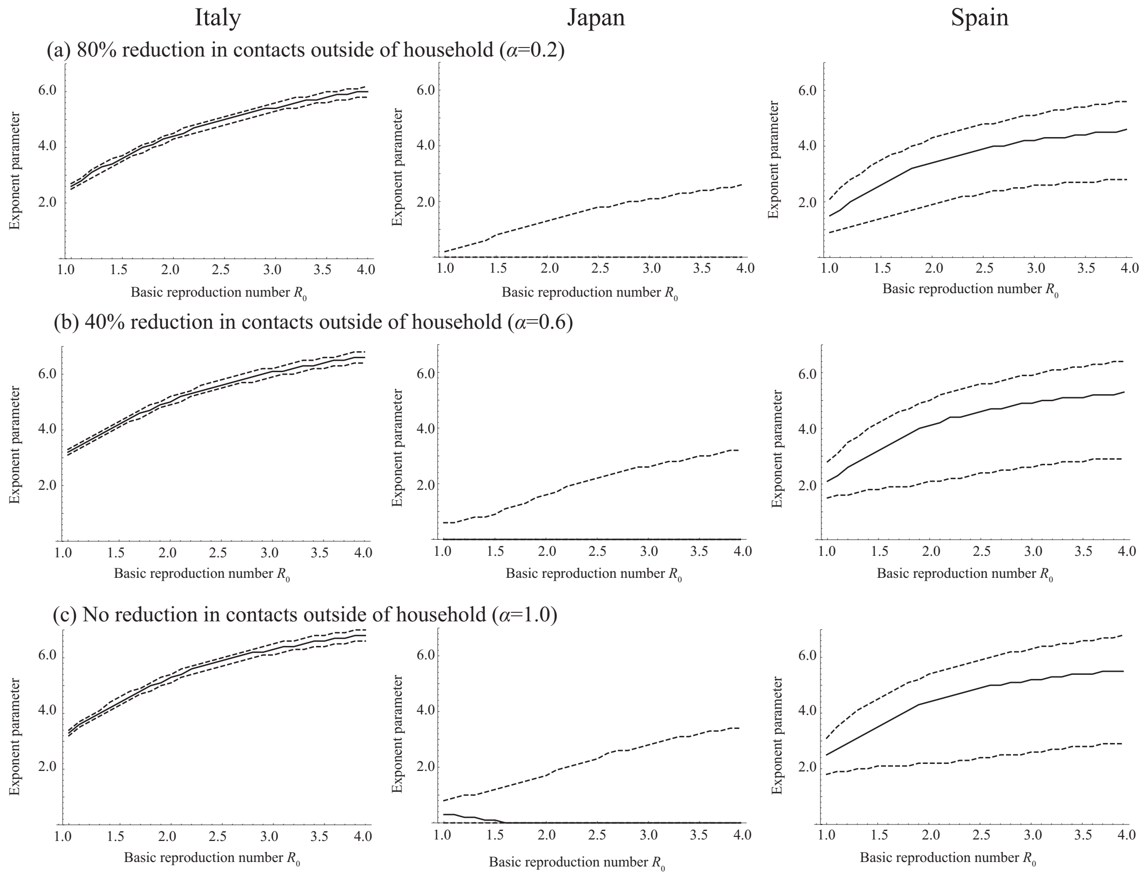

Figure s2: The estimate of exponent parameter φ describing the variation of susceptibility among age groups using model 2 and assuming that the fraction of infections that becomes symptomatic among all COVID-19 cases is 0.05. True and broken lines represent the maximum likelihood estimates and 95% confidence intervals, respectively.

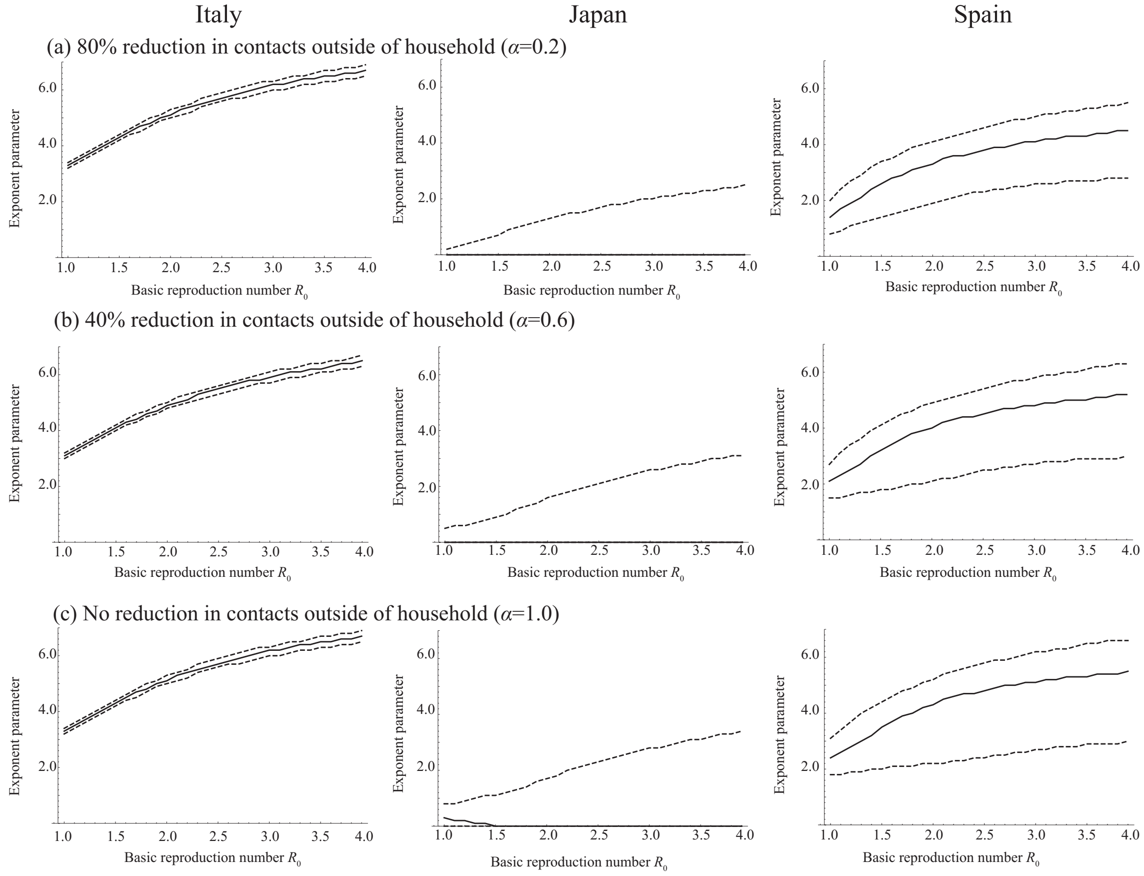

Supplemental text

The proof of proportionality between the number of mortality and recovered individuals in model 1

We consider the following special setting

$$
\begin{aligned}
S_n'(t) &= -\sigma_n \beta \sum_m k_{n,m} I_m(t) S_n(t), \\
E_n'(t) &= \sigma_n \beta \sum_m k_{n,m} I_m(t) S_n(t) - \epsilon E_n, \\
I_n'(t) &= \epsilon E_n - (\gamma + \delta) I_n, \\
R_n'(t) &= \gamma I_n, \\
D_n'(t) &= \delta I_n,
\end{aligned}
$$

where $n \in \{1,2,\dots,\}$ represents the age-class. We refer to the main text for parameter descriptions. Here it is assumed that the mortality rate δ is age-independent.

From the above equations, it is straightforward to see that

$$D_n(t) = \delta \int_0^t I_n(s) ds,\ R_n(t) = \gamma \int_0^t I_n(s) ds.$$

Thus

$$\frac{D_n(t)}{\sum_j D_j(t)} = \frac{R_n(t)}{\sum_j R_j(t)} = \frac{\int_0^t I_n(s) ds}{\sum_j \int_0^t I_j(s) ds},$$

which implies that the age-distribution of deaths is determined by the age-distribution of the recovered population, assuming that the mortality rate δ is age-independent.

Next we vary only parameters β and δ fixing $\frac{\beta}{\gamma+\delta}$. We show that this parameter configuration does not affect the age-distribution. We show that for a fixed $\frac{\beta}{\gamma+\delta}$ and given elements of contact matrix $k_{n,m}$, and progression rates ϵ and γ, the final distribution

$$\lim_{t\to\infty}\frac{R_n(t)}{\sum_j R_j(t)} = \lim_{t\to\infty}\frac{D_n(t)}{\sum_j D_j(t)},\ n \in \{1,2,\dots\}$$

is uniquely determined. To this aim, we introduce the nondimensionalized time, $\tau = (\gamma + \delta)t$. Denoted

$$\tilde{S}_n(\tau) = S_n(t),\ \tilde{E}_n(\tau) = E_n(t),\ \tilde{I}_n(\tau) = I_n(t),\ \tilde{R}_n(\tau) = R_n(t),\ \tilde{D}_n(\tau) = D_n(t).$$

Dropping tilde, we have

$$\begin{aligned}
S_n'(\tau) &= -\frac{\beta}{\gamma+\delta}\sum_m k_{n,m} I_m(\tau)S_n(\tau)\\
E_n'(\tau) &= \frac{\beta}{\gamma+\delta}\sum_m k_{n,m} I_m(\tau)S_n(\tau) - \frac{\epsilon}{\gamma+\delta}E_n\\
I_n'(\tau) &= \frac{\epsilon}{\gamma+\delta}E_n - I_n\\
R_n'(\tau) &= \frac{\gamma}{\gamma+\delta}I_n\\
D_n'(\tau) &= \frac{\delta}{\gamma+\delta}I_n.
\end{aligned}$$

From the fundamental theory of differential equations, the model has a unique solution with a suitable initial condition. Thus, the nondimensionalized model shows that, for a fixed $\frac{\beta}{\gamma+\delta}$ and given elements of contact matrix $k_{n,m}$, and progression rates ϵ and γ, the final distribution of deaths is uniquely determined.
